# Internal Fixation Method Using EZ-Tcon for Transconjunctival Fat Repositioning: Clinical Outcomes and Efficacy

**DOI:** 10.1007/s00266-020-01873-1

**Published:** 2020-07-21

**Authors:** Cheol Ho Chang, Juyoung Bae, Myung Kyu Cha, Sa Ik Bang, Kyeong-Tae Lee

**Affiliations:** 1 The Wannabe Plastic Surgical Clinic, Seoul, Korea; 2 Department of Plastic Surgery, Samsung Medical Center, Sungkyunkwan University School of Medicine, 81 Irwon-ro, Gangnam-gu, Seoul, 06351 South Korea

**Keywords:** Tear trough deformity, Transconjunctival fat repositioning, Tnternal fixation, Complication

## Abstract

**Background:**

Transconjunctival fat repositioning is the gold standard for the correction of tear trough deformity. For fixation of fat pedicle, the internal fixation (IF) and externalized percutaneous suture (EPS) techniques are used, which have their own advantages and disadvantages. The present study aimed to introduce a new IF technique using a devised needle (EZ-Tcon) and to compare its outcomes with those of the conventional EPS technique.

**Methods:**

Patients with primary tear trough deformity who underwent transconjunctival fat repositioning were reviewed and categorized into two cohorts according to the fixation technique: cohort 1 consisted of patients treated using the conventional EPS technique and cohort 2 consisted of those in whom the new IF technique using EZ-Tcon was adopted. Post-operative complications and aesthetic outcomes were assessed using a four-scale grading system.

**Results:**

A total of 545 patients, 211 from cohort 1 and 344 from cohort 2 were evaluated with a median follow-up of 70 days. Compared to cohort 1, cohort 2 showed significantly lower rates of long-standing conspicuous scars on lower eyelid, re-operation and overall complications. In the analysis of aesthetic outcomes, 88.9 percent of cohort 2 showed grade 0 (no deformity) or I (mild deformity) post-operatively. The rate of excellent outcomes (improvements of ≥ two grades) was significantly higher in cohort 2 than in cohort 1 (*p*-value < 0.001).

**Conclusion:**

Our technique using EZ-Tcon could possess advantages of the conventional IF and EPS techniques, showing lower complication rates and aesthetically satisfactory outcomes, and could be a safe and reliable method of transconjunctival fat repositioning.

**Level of Evidence IV:**

**T**his journal requires that authors assign a level of evidence to each article. For a full description of these Evidence-Based Medicine ratings, please refer to the Table of Contents or the online Instructions to Authors www.springer.com/00266.

**Electronic supplementary material:**

The online version of this article (10.1007/s00266-020-01873-1) contains supplementary material, which is available to authorized users.

## Introduction

Tear trough deformity represents a set of conditions showing a prominent nasojugal groove with the medial periorbital hollow that extends obliquely from the medial canthus. It can be caused by a bulge of the orbital fat through the attenuating orbital retaining ligament and sagging of the orbicularis oculi muscle as well as through reduction of the volume of facial fat compartments [1, 2]. Diverse treatment modalities have been attempted for its correction [3], for the purpose of seeking an ideal method that can provide long-lasting aesthetically pleasing outcomes with minimal violation of the normal anatomy. Among them, transconjunctival repositioning of the orbital fat along the infraorbital rim, first introduced by Loeb [4] and further modified by many surgeons including Goldberg [5], has been popularly used, and it is considered as the gold standard for correction of tear trough deformity [5–7].

**Fig. 1:**
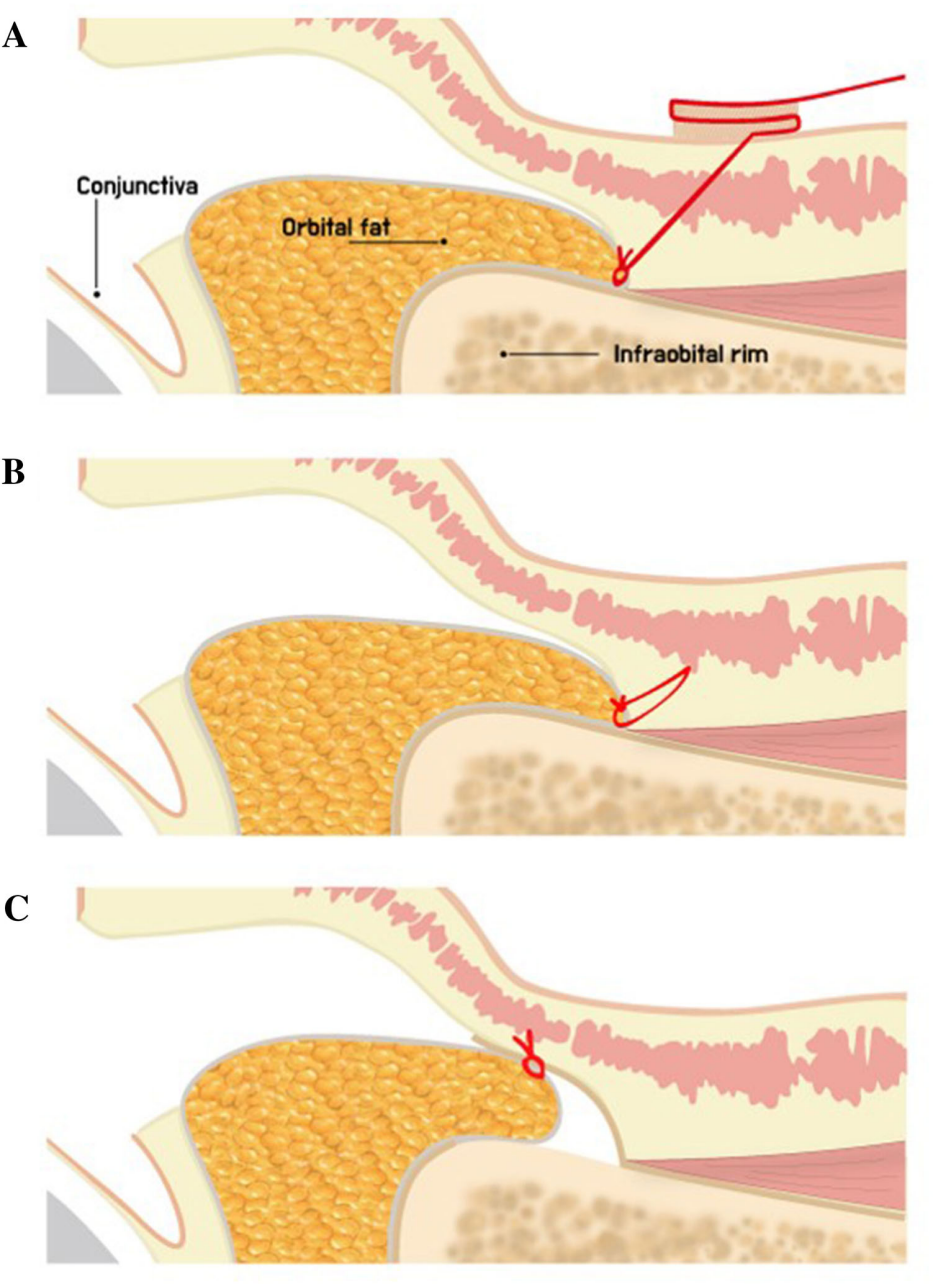
Fixation position of transposed fat pedicles. Orbital fat can be secured at the lowermost end of the dissected space with the externalized percutaneous suture technique **(a)** and internal fixation technique by EZ-Tcon **(b)**, whereas fixation may be conducted at a more cephalic position with the conventional internal fixation technique owing to limited working space **(c)**

With an effort to achieve optimal outcomes, there has been a debate on the technical specifications of transconjunctival fat repositioning, which include dissection plane (subperiosteal vs. supraperiosteal) for pocket preparation [8] and methods of fat repositioning (redraping of pedicled fat versus repositioning with septal reset) [9]. Selection of fixation methods for transposed fat pedicles has been one of the main issues. Traditionally, two methods have been commonly used for fixation of transposed fat: externalized percutaneous suture (EPS) technique and internal fixation (IF) technique. Each technique has its own advantages and disadvantages. The main advantage of the EPS technique is technical ease, which allows the use of a shorter incision and enables fixation of fat into the optimally lowermost position in the pocket without difficulty. Further, it can be performed in the supraperiosteal or subperiosteal planes. However, this method is limited by a relatively higher chance of relapse owing to less secure fixation. Besides, post-operative discomfort related with pull-out suture is one of the main drawbacks of this method, which requires additional visits to the outpatient clinic for suture removal and can often result in local inflammation or dermatitis. Meanwhile, the IF technique can provide secure and rigid fixation, leading to a relatively lower rate of relapse theoretically, and does not cause problems related to fixation of sutures. However, this method can be technically demanding owing to a narrow operation field, needing a relatively steep learning curve, and often require a longer incision. Its use can be limited in the subperiosteal plane only for firm anchoring. In addition, there could be a chance of fixation at a less optimum position, especially for a novice, which can result in unfavourable results (Fig. 1).

**Fig. 2:**
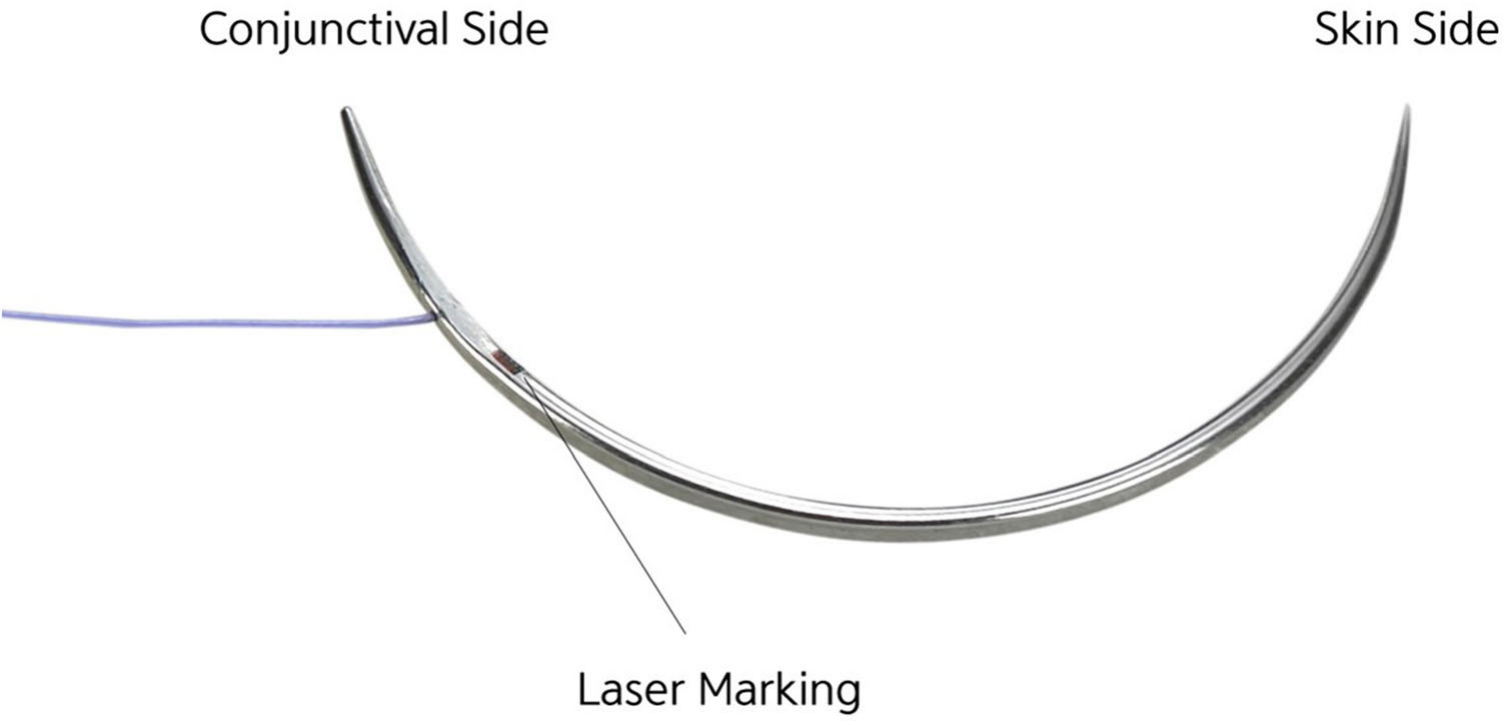
Picture of EZ-Tcon. EZ-Tcon is composed of Chang’s needle and absorbable thread connected with it. Chang’s needle is 28 mm in length, 3/8 circle, and bidirectional type. The skin side end is sharp round or cutting style for easy skin penetration, whereas the conjunctival side end is blunt round style to reduce the chance of blood vessels and eyeball injury. There is a laser mark from the thread attachment point for easy prediction of thread’s position from skin side to facilitate prevention of skin dimpling caused by unintentional exposure of thread

In 2017, the author introduced a new internal fixation technique using a devised needle, termed as “Chang’s needle”, for transconjunctival fat repositioning [10]. EZ-Tcon (HandBioMed Corp, Korea) is the commercial name of Chang’s needle attached with 4–0, 5–0 and 6–0 absorbable thread (polyglycolic acid or polyglycolide-co-caprolactone) (Fig. 2). This technique was suggested to have the strengths of both IF and EPS techniques theoretically, including technical ease, rigid fixation, elimination of additional visit for removal of pull-out suture and reliable and aesthetically pleasing outcome with a low relapse rate. However, whether these theoretical strengths of this method could lead to practical benefits and efficacy in the clinical settings remains unclear. Therefore, the present study aimed to compare the clinical outcomes of our new IF method for transconjunctival fat repositioning with those of the conventional EPS method.

## Patients and Methods

### Study Population

Patients who presented with a primary tear trough deformity and were treated with transconjunctival fat repositioning by a single surgeon at the Wannabe Plastic Surgical Clinic between January 2010 and May 2019 were retrospectively reviewed. The patients were excluded if they visited for revision operation after they had undergone a previous operation at other clinics, or had been lost to follow-up within one month after the operation.

The patients were categorized into two cohorts according to the fixation method of transposed fat. Cohort 1 consisted of patients who were treated with the conventional EPS technique, between January 2010 and July 2016. This cohort served as the control in the following analysis. Cohort 2 included patients who were treated with the new IF technique using the devised needle EZ-Tcon from August 2016 to May 2019.

### Surgical Procedure

All operations were conducted under local anaesthesia. In the control group (Cohort 1), the conventional EPS technique was used. Transconjunctival incision was made using electrocautery 3–4 mm above the fornix, approximately 12 mm in length. Dissection was performed along the orbital septum until the arcus marginalis was reached. Then, supraperiosteal dissection was performed 10–12 mm in vertical length from the arcus marginalis, freeing the tear trough ligament and palpebromalar ligaments from the underlying tissue. After exposing the orbital fat pad, fat pedicle was prepared by careful dissection of the surrounding fibrous tissues. The prepared orbital fat was transposed into the supraperiosteal pocket and fixed with externalized loops of 5–0 absorbable sutures (See video, Supplemental Digital content 1). The pull-out threads were removed after five to six days post-operatively [2, 9]. (See video, Supplemental Digital content 2).

**Fig. 3:**
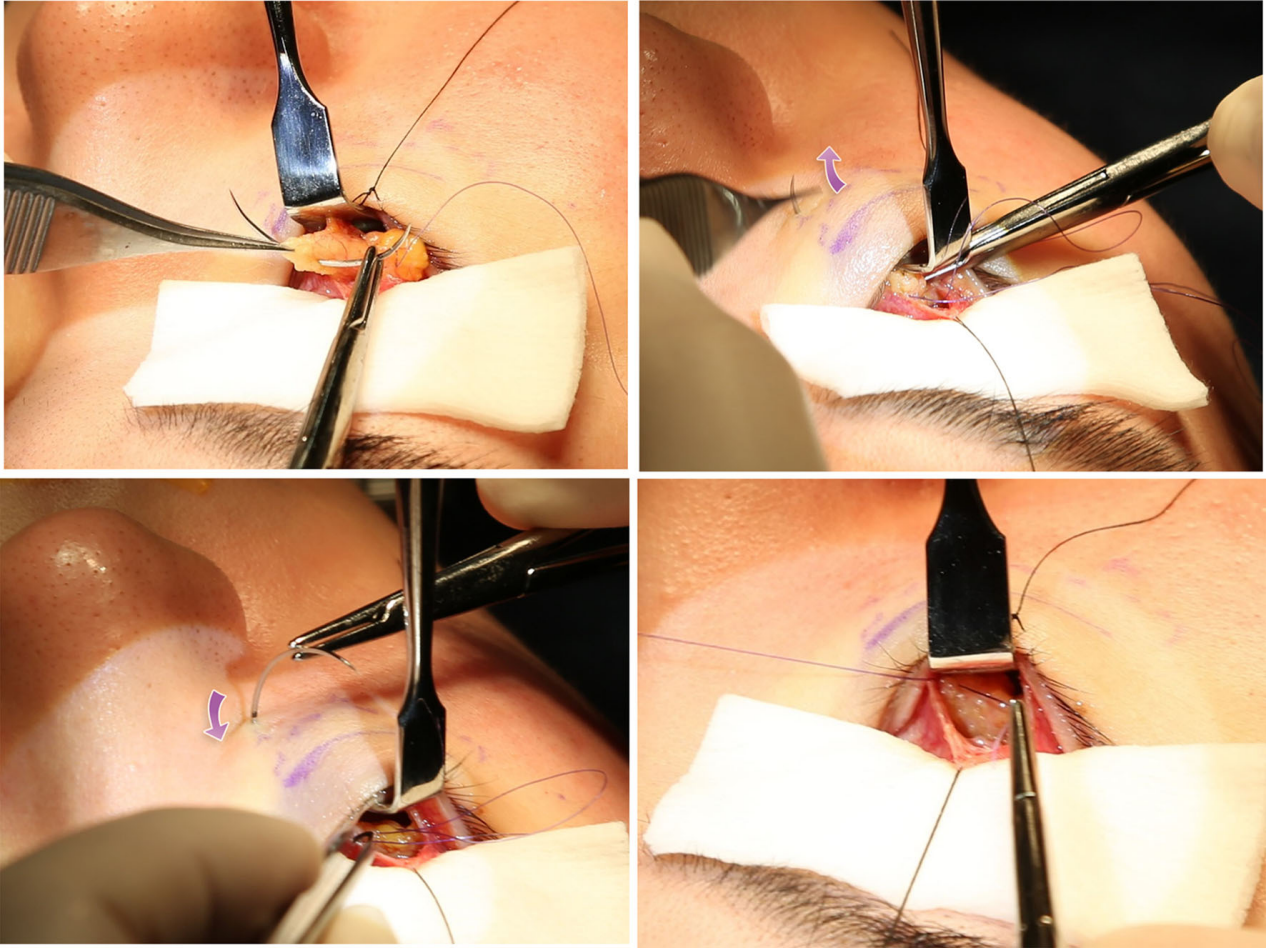
The internal fixation technique using EZ-Tcon. The devised needle is placed at the fat pedicle (above, left). EZ-Tcon is passed from the distal end of the dissected space to the skin with paying attention to not pulling it beyond the laser mark (above, right). After checking the laser mark, the needle is passed back into the supraperiosteal dissected space, which is 2-3 mm from the original puncture point (below, left). As a result, part of the absorbable thread is trapped in the premaxillary soft tissue, and the transposed orbital fat can be fixed to the lowermost dissected space by placing several knots

**Fig. 4:**
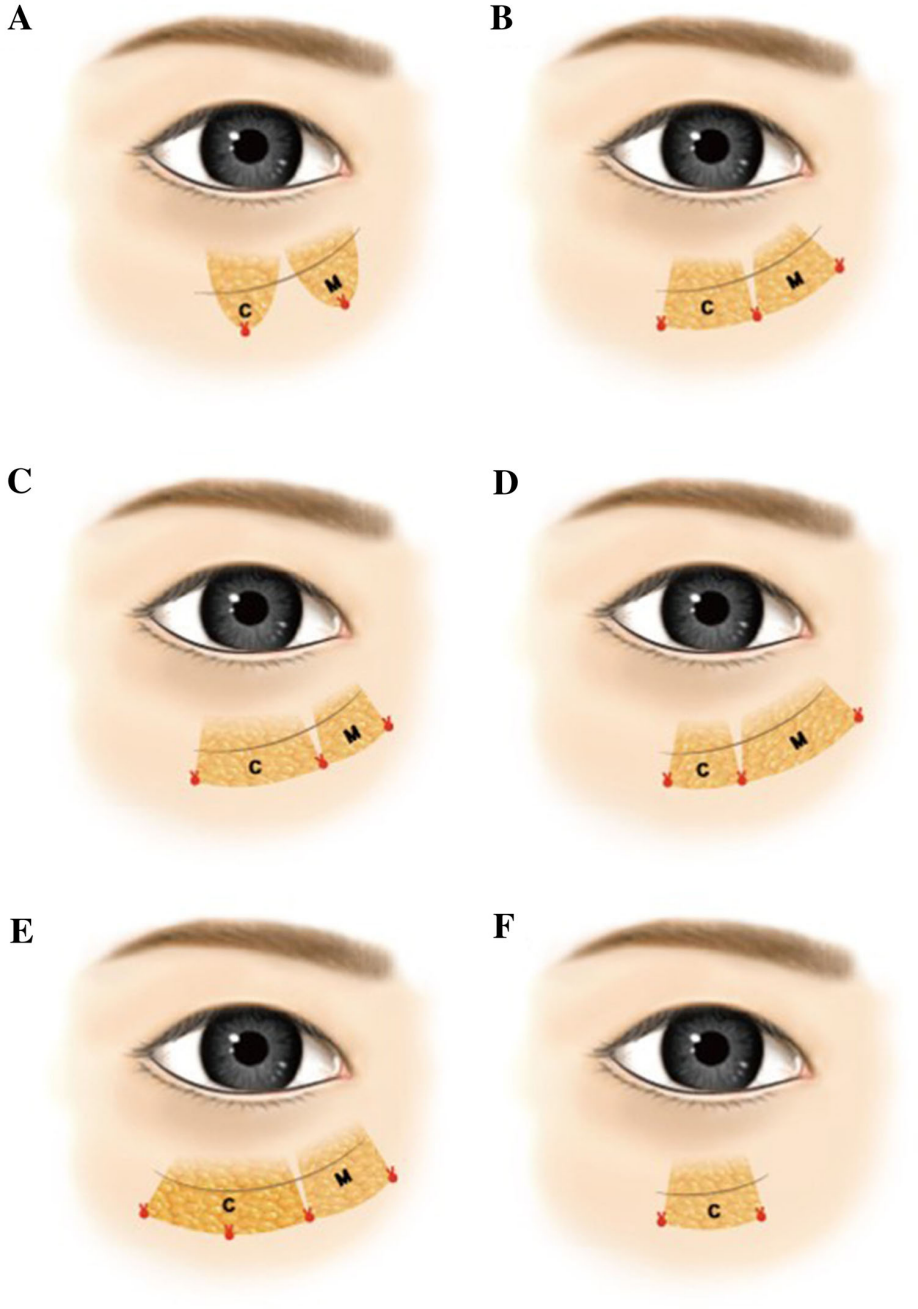
Fixation style of orbital fat pedicles. Two-point fixation for medial and central fat pedicle, respectively, which could cause contour irregularity **(a)**. Three-point fixation enables even distribution of medial and central orbital fat **(b)**. When the volume of central fat exceeds that of medial fat, medialization of the 2nd fixation point can be performed, which provides wider coverage by the central fat pedicle and eventually more even volumetric distribution **(c)**. Reverse case of C **(d)**. In a case of long tear trough line in Grade II or III in modified Barton’s grading system, additional suture in the midpoint of central fat is useful for lowermost fixation over the full length**(e)**. If there is only lateral depression (Grade 1_L_ in the modified Barton’s grading system), the two points fixation of central fat pedicle would be sufficient for coverage **(f)**

The new IF technique used in Cohort 2 was conducted as previously reported (Fig. 3) [10]. In brief, supraperiosteal pocket dissection and fat pedicle preparation were performed in the same way as in the conventional EPS technique described above. The skin end of 5–0 EZ-Tcon was penetrated through the prepared fat pedicle and passed from the lowermost point of the dissected space to the skin, until the laser marking on it was seen from the skin side or until its conjunctival end is not visible from the conjunctival side. Then, the needle was passed back into the dissected space to approximately 2–3 mm from the previous entry point of the needle, which traps some portion of the thread at the premaxillary soft tissue. Then, inner fixation was completed by tie (See video, Supplemental Digital content 3). Fixation was performed at three points, which could be adjusted to two or four points individually according to the distribution pattern of orbital fat (Fig. 4). A single 6–0 absorbable suture was placed at the centre of wound. A light dressing was usually applied using hydrocolloid to absorb slight oozing from the needle puncture site and to reduce post-operative swelling; the dressing was removed by the patients themselves the next day.

For middle-aged patients with excessive skin laxity and wrinkles on the lower eyelid, lateral partial incision blepharoplasty was conducted in both groups, which consisted of excision of redundant skin and suspension of the orbicularis oculi muscle.

### Outcome Measure

The primary outcomes were rates of post-operative complications and aesthetic results. Post-operative complications included hematoma, infection, granuloma, long-lasting (> 4 weeks) conspicuous scars on the lower eyelid, ectropion, retraction, diplopia, chronic chemosis over two weeks post-operatively, and reoperation. The long-lasting conspicuous scars were defined as scar conditions visible after four weeks, which due to needle penetration or placement of sutures. Cases with reoperation were defined as those undergoing take-back to the operating room due to the development of complications, including haematoma or infection.

Aesthetic outcomes were assessed using a four-scale grading system, ranging from 0 to III, as shown in Table 1. This was modified based on Barton’s grading system [11] by adding a new I_L_ grade that was characterized by the mild, subtle presence of a lateral line or shadow and the absence of a medial line. The patient photographs taken by professional photographers at pre-operative and final post-operative visits were used for the evaluation. Two plastic surgeons who were not involved in the operations participated in the evaluation of the aesthetic outcomes, with the final decision based on discussions in case of any disagreements. They were blinded to which surgical group the patients belonged to. In addition to the distribution of pre-operative and post-operative grades, the rates of cases showing excellent results and improvement/no improvements were also evaluated. Excellent outcomes were defined as improvements of ≥ two grades at the final follow-up photograph compared to the pre-operative appearance. Cases with no improvement were defined as those showing the same grade post-operatively and pre-operatively.

**Table 1:** Modified Barton’s grading system

Grade	Anatomic analysis
0	The absence of medial or lateral lines demarcating the arcus marginalis or the orbital rim, and a smooth youthful contour without a transition zone at the orbit–cheek junction
I_M_	Mild, subtle presence of a medial line or shadow; smooth lateral transition of lid–cheek junction
I_L_	Mild, subtle presence of a lateral line or shadow; The absence of a medial line
II	Moderate prominence of a visible demarcation of the lid–cheek junction extending from medial to lateral
III	Severe demarcation of the orbit–cheek junction with an obvious step between the orbit and the cheek

Grade I_L_ is newly added to the original grading system

### Statistical Analysis

The patient demographics, complication profiles and aesthetic outcomes were compared between the two cohorts. Pearson’s Chi-square test or Fisher’s exact test was used for analysis of the categorical variables, and the Student’s *t*-test or Mann–Whitney test for that of continuous variables. To identify independent predictors for the development of complications and excellent outcomes, univariable and multivariable logistic regression analyses were conducted by calculating odds ratio (OR) and 95% confidence intervals (CIs). A p-value of less than 0.05 was considered statistically significant. All statistical analyses were conducted using IBM SPSS 20.0 (IBM Corp., Armonk, NY.).

## Results

A total of 1,119 patients with tear trough deformity who underwent transconjunctival fat repositioning were identified at the initial search. After the application of the above selection criteria, 545 patients who were followed up for at least post-operative one month were included in the final analysis. The mean age was 34.2 years. There were 59 male and 486 female patients. The median follow-up period of the overall study population was 70 days.

Of 545 patients, 211 were included in cohort 1 and 334 were included in cohort 2. The patients in cohort 2 tended to be younger and included more male patients compared to those in cohort 1, which showed no statistically significant differences (Table 2). The rate of performing lateral partial incision blepharoplasty was significantly higher in cohort 2 (30.5% vs. 22.7%). Regarding the pre-operative grade of tear trough deformity, the two groups had similar distribution (*p*-value = 0.364), showing patients with grade II and III in approximately 95 percent in both.

**Table 2:** Patient demographics and pre-operative grade of tear trough deformity in two cohorts

	Overall	Cohort 1 (*n* = 211)	Cohort 2 (*n* = 334)	*p*-value
Demographics				
Age (mean ± SD)	34.2 (± 11.8)	35.2 (± 10.9)	33.6 (± 12.4)	0.113
Sex				0.053
Male	59	16 (7.6%)	43 (12.9%)	
Female	486	195 (92.4%)	291 (87.1%)	
Follow-up period (days), median	70	58	73	0.002
Pre-op grade of tear trough deformity				0.364
0	1	0	0	
I	20	11 (5.2%)	10 (3.0%)	
I_M_	14	8 (3.8%)	6 (1.8%)	
I_L_	7	3 (1.4%)	4 (1.2%)	
II	294	115 (54.5%)	179 (53.6%)	
III	230	85 (40.3%)	145 (43.4%)	

### Post-operative Complications

**Fig. 5:**
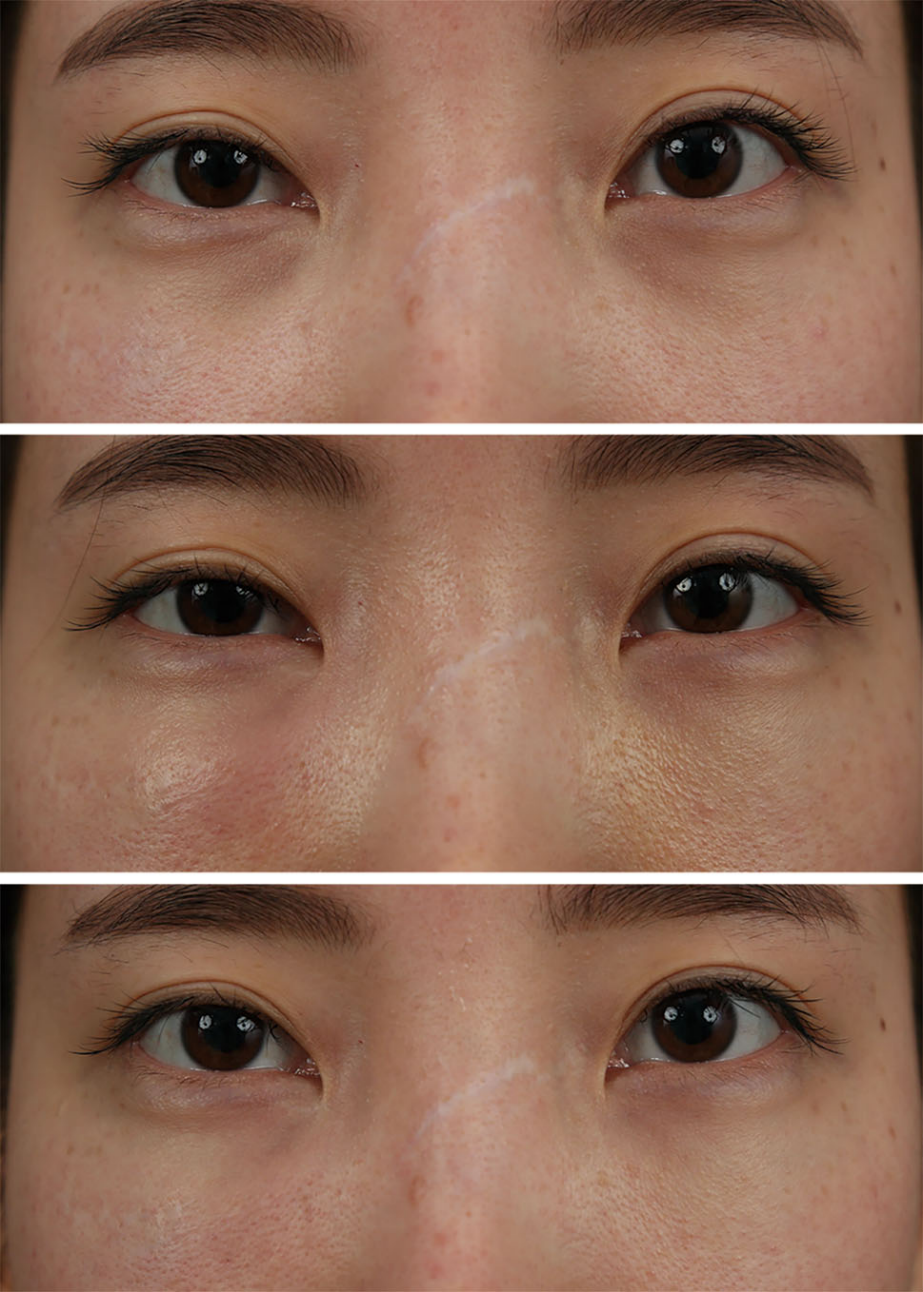
Development of local infection following a transconjunctival fat repositioning with internal fixation technique using EZ-Tcon. Pre-operative appearance (Above). Occurrence of focal redness, swelling and tenderness on her right lower eyelid at post-operative 5 days (Middle). The lesion was resolved with conservative managements using intravenous antibiotics administration for three days (Below)

Table 3 lists the complication profiles of the two groups. Post-operative complications developed in 29 patients (5.3%) during the follow-up period. None of them developed ectropion, retraction and chronic chemosis. Reoperation was conducted in eight patients owing to complications including haematoma (*n* = 7) and infection (*n* = 1). All patients with haematoma underwent reoperation for removal regardless of its extent to prevent fibrosis and retraction. Of two patients with infection, one in cohort 1 underwent take-back and underwent drainage of pus and irrigation with antibiotic solutions through the conjunctival wound. Another patient in cohort 2 was managed with conservative treatment including intravenous antibiotics (Fig. 5). Two patients in cohort 2 complained of palpable granuloma of approximately 2–3 mm in diameter, which was treated with diluted triamcinolone injection. Transient diplopia, which developed in one case (cohort 1), was spontaneously resolved within one-month post-operatively. The rate of overall complications was significantly lower in cohort 2 than in cohort 1. Especially, cohort 2 had significantly lower rates of conspicuous scars and reoperation than cohort 1. The rates of other complications including infection did not differ between the cohorts. Multivariable analysis demonstrated that the type of fixation method had a significant influence on the development of overall complications, showing that cohort 2 had significantly lower rates of complications than cohort 1 after adjusting for other variables (OR; 0.102, 95% CI; 0.038–0.280, *p*-value < 0.001). Other variables including sex, age and lateral partial incision blepharoplasty were not influence significantly.

**Table 3:** Comparison of complication profiles between cohort 1 and cohort 2

Complication profiles	Overall	Cohort 1	Cohort 2	*p*-value
Overall complications	29	24 (11.4%)	5 (1.5%)	< 0.001
Haematoma	7	5 (2.3%)	2 (0.6%)	0.074
Infection	2	1 (0.3%)	1 (0.3%)	0.743
Granuloma	2	0	2 (0.6%)	0.260
Conspicuous scars on lower eyelid	17	17 (8.1%)	0	< 0.001
Diplopia	1	1 (0.3%)	0	0.208
Reoperation	8	6 (2.8%)	2 (0.6%)	0.034

### Aesthetic Outcomes

In the evaluation of post-operative aesthetic outcomes, 514 (94.3%) out of 545 patients showed improvements. Specifically, a majority of patients showed grade 0 (33.8%) and grade I (49.4%) deformity post-operatively. Only 31 patients (5.7%) showed no improvement. The rate of no improvement was significantly lower in cohort 2 than in cohort 1 (3.3% vs. 9.5%, *p*-value = 0.002). Excellent outcomes with improvement of ≥ 2 grades were observed in 266 patients (48.8%) overall. Cohort 2 showed significantly higher rate of excellent outcomes than cohort 1 (Table 4).

**Table 4:** Comparison of post-operative aesthetic outcomes between two cohorts

	Overall	Cohort 1	Cohort 2	*p*-value
Post-op grade of tear trough deformity				< 0.001
0	184 (33.8%)	35 (16.6%)	149 (44.6%)	
I	269 (49.4%)	121 (57.3%)	148 (44.3%)	
II	86 (15.8%)	50 (23.7%)	36 (10.8%)	
III	6 (1.1%)	5 (2.4%)	1 (0.3%)	
Aesthetic outcomes				0.002
Improvement	514 (94.3%)	191 (90.5%)	323 (96.7%)	
No improvement	31 (5.7%)	20 (9.5%)	11 (3.3%)	
Excellent outcomes	266 (48.8%)	70 (33.2%)	196 (58.7%)	< 0.001

Multivariable analysis showed that the fixation method was an independent predictor for excellent outcomes, showing that cohort 2 had significantly higher odds for achieving excellent outcomes than cohort 1 (OR; 3.365, 95% CI; 2.232–5.073, *p*-value < 0.001). The patient sex and pre-operative aesthetic grade also significantly influenced the outcome (Table 5).

**Table 5:** Multivariable analyses for independent predictors of achieving excellent outcomes

Variables	Adjusted *p*-value	OR (95% CI)
Sex		
Male	Ref	
Female	0.044	1.927 (1.018 – 3.648)
Age	0.344	1.013 (0.987 – 1.039)
Lateral partial incision blepharoplasty	0.404	1.335 (0.677 – 2.634)
Fixation method		
EPS (cohort 1)	Ref	
IF (cohort 2)	< 0.001	3.365 (2.232 – 5.073)
Pre-operative grade		
Grade II versus I	0.334	1.692 (0.581 – 4.925)
Grade III versus I	< 0.001	7.803 (2.641 – 23.054)

**Fig. 6:**
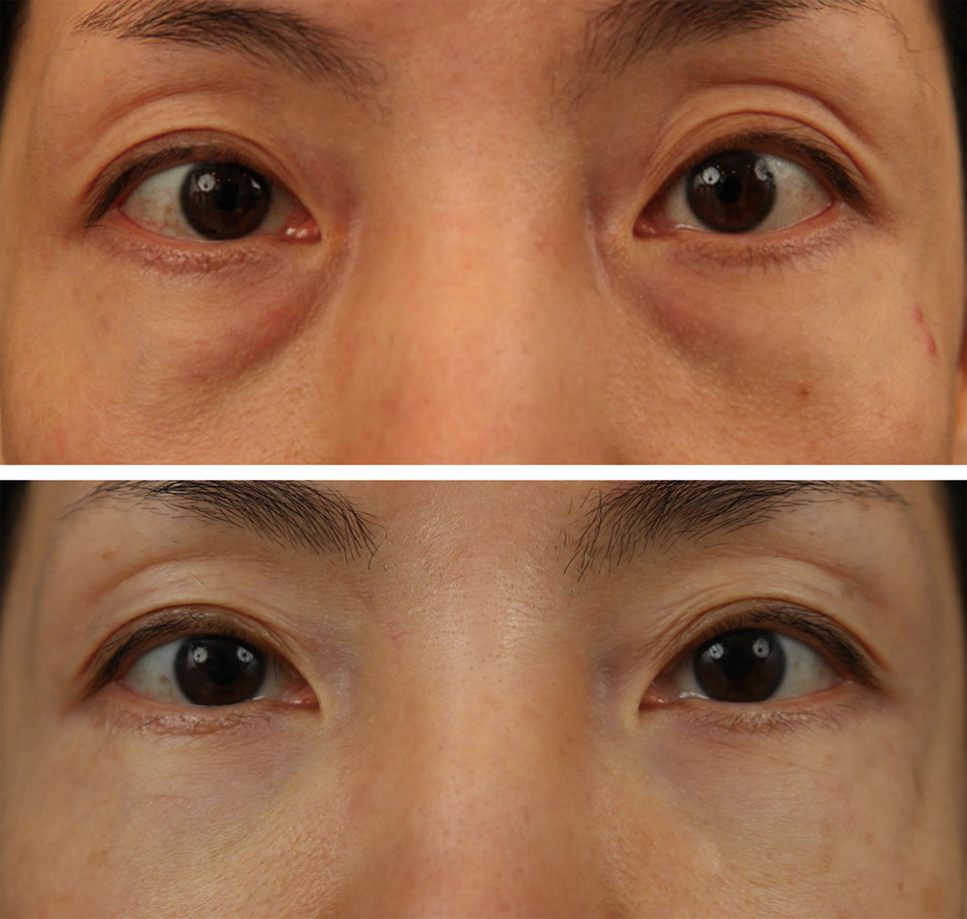
Case 1. A 41-year-old woman visited our clinic for correction of a tear trough deformity of grade I_M_ (Above). She underwent the transconjunctival fat repositioning using the externalized percutaneous suture technique with lateral partial incision blepharoplasty (Cohort 1). Appearance at post-operative eight years, showing excellent long-term outcomes without recurrence (Grade 0) (Below)

Representative cases are shown in Figs. 6 (Cohort 1), 7 and 8 (Cohort 2).

**Fig. 7:**
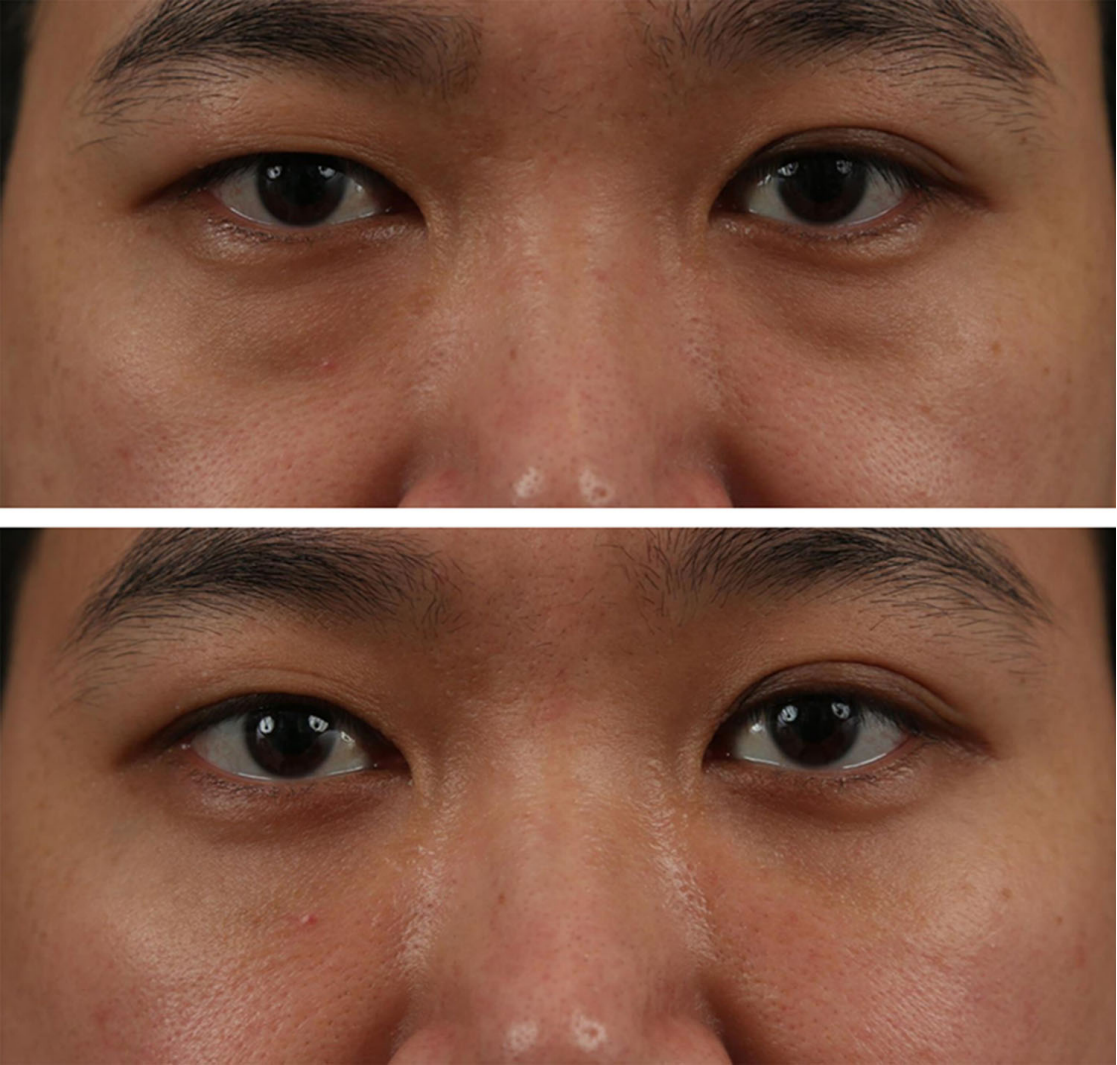
Case 2. A 35-year-old man presented a tear trough deformity of Modified-Barton grade I_L_ (lateral mild groove without medial depression) (Above). He was treated with transconjunctival fat repositioning with internal fixation technique using EZ-Tcon (Cohort 2). Appearance at post-operative 3 months (Below)

**Fig. 8:**
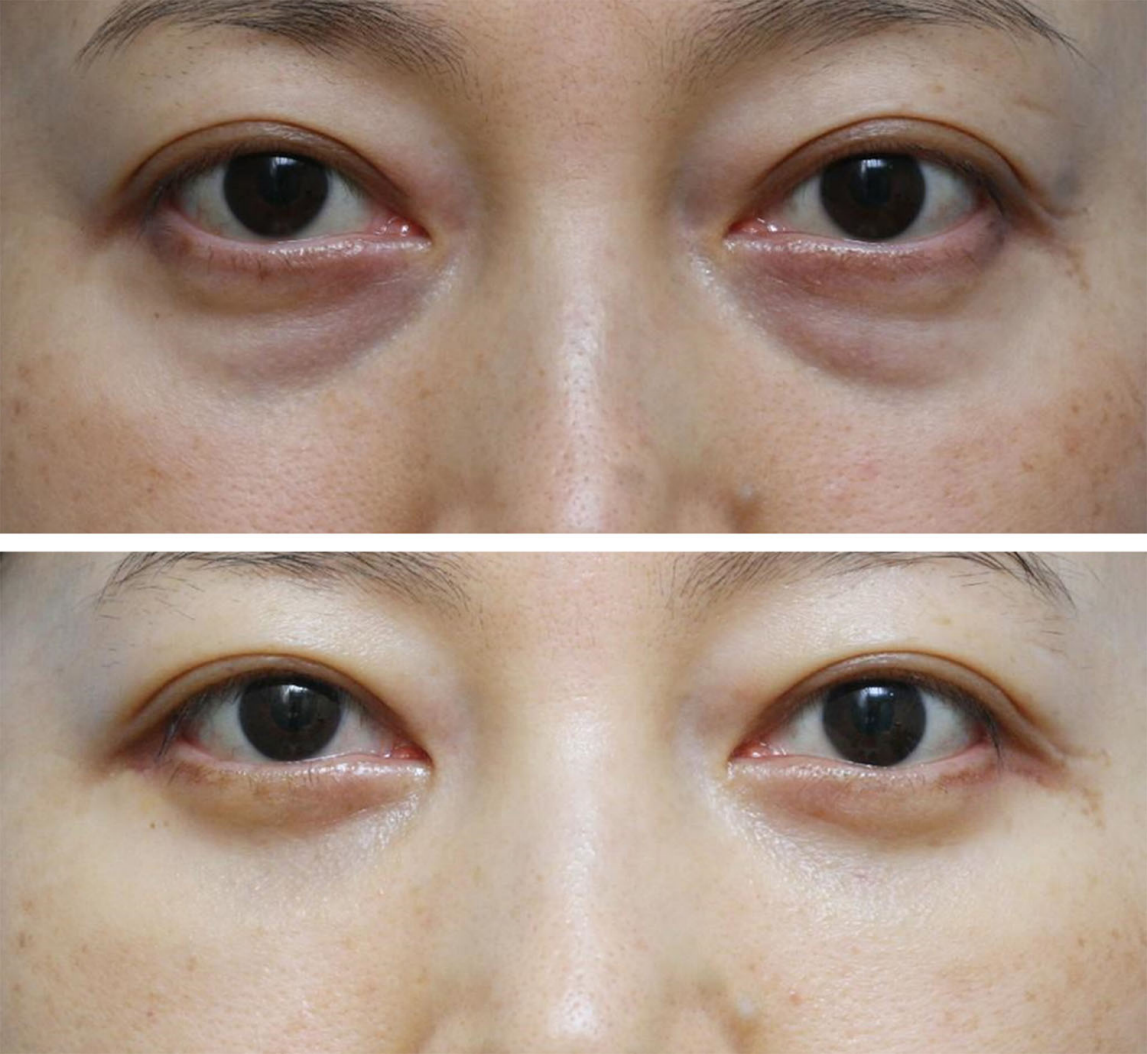
Case 3. A 44-year-old woman had tear trough deformity of modified Barton Grade II and mild skin laxity (Above). She underwent transconjunctival fat repositioning using EZ-Tcon and lateral partial incision blepharoplasty (Cohort 2). Tear trough deformity was improved to grade 0 with preservation of the pretarsal roll which can make her look younger and healthier (Below)

## Discussion

The presents study described in detail a new IF technique using the devised needle EZ-Tcon for correction of tear trough deformity and evaluated its clinical outcomes with a considerable number of cases. Besides, a comprehensive head-to-head comparison of the outcomes between the new IF technique and popularly used conventional EPS technique was conducted not only in terms of complications, but also in aesthetic outcomes.

**Fig. 9:**
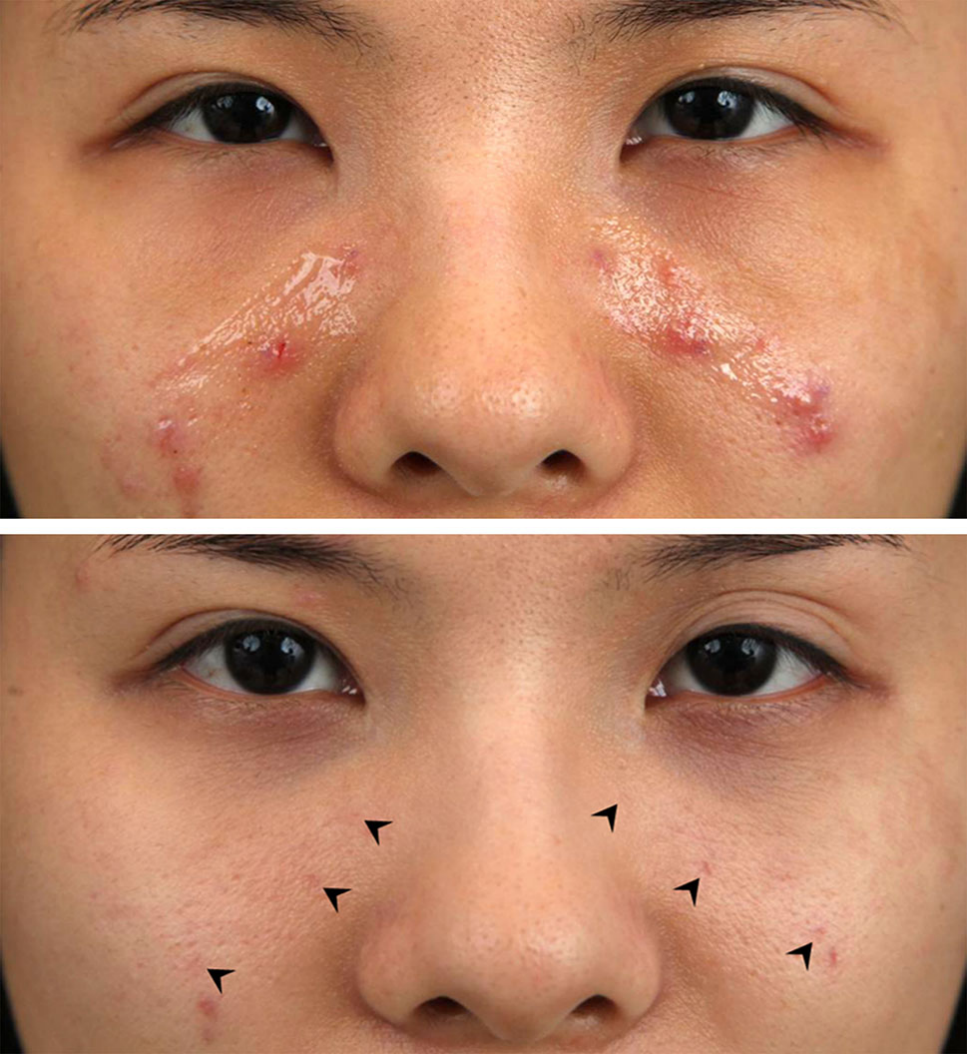
After removal of thread and taping in cases using the EPS technique, skin inflammation and dermatitis around the site of thread/needle penetration are observed (Left), which could last after post-operative 5 weeks (Right)

We found that cohort 2 showed a significantly lower rate of post-operative complications than cohort 1. In particular, the rate of long-lasting conspicuous scars on the lower eyelid had the greatest difference showing 8.1 percent in cohort 1 and 0 percent in cohort 2. The presence of sutures for five or six days could cause local inflammation around the stitches especially in cases with oily and acneiform skin, which can lead to long-lasting stitch mark (Fig. 9). Further, prolonged taping of the skin for fixing threads can cause skin complications or contact dermatitis to sensitive skin. This could distress patients and eventually reduce the overall satisfaction. With our new IF technique, this skin problem and long-lasting scars, one of the common complaints related to the EPS technique, can be avoided as shown here. In addition, this allows for earlier recovery to social life and daily living, as application of taping for longer period is not necessary.

In the current study, significantly better aesthetic outcomes were observed in patients in cohort 2 comparted to those of cohort 1. Specifically, cohort 2 showed significantly fewer cases with no improvement and significantly more with excellent outcomes than cohort 1. Theoretically, to achieve aesthetically optimal results, two factors are required to be met: transferring the orbital fat pedicle into the proper position and fixating it rigidly to avoid reverting to the original position after a long time. Regarding the former, the fat pedicle should be transferred and fixed to the lowermost portion of the prepared pocket. However, with the conventional IF technique, placing fixation sutures at the lowermost position is technically demanding owing to the narrow transconjunctival incision; therefore, fixating at a less optimum position often occurs, especially for a novice surgeon, which can result in suboptimal outcomes. To avoid this adverse result, lengthening of transconjunctival incision can often be conducted, which could cause more frequent chronic chemosis and patient discomfort after operation. However, with our new IF technique using EZ-Tcon, these limitations of the conventional method can be easily overcome. All procedures of the IF technique were conducted through transconjunctival incision of approximately 12 mm in length, without lengthening the incision. Moreover, no patients showed chronic chemosis post-operatively. Excellent aesthetic outcomes with approximately 90 per cent of patients from cohort 2 showing grade 0 or I post-operatively further support the technical feasibility of our procedure for fixation lowermost position of the fat pedicle. It can be assumed that this technique using EZ-Tcon can allow for more facile internal fixation of transposed fat into the lowermost position with short incision, which can lead to achieving optimal outcomes and reducing morbidity compared to those obtained using the conventional IF technique.

Durability of fixation of transferred fat is another critical factor to determine final aesthetic outcomes, as mentioned above. The EPS technique is popular owing to technical ease and reliability for fixation of the fat pedicle into an optimal position, which can satisfy the first condition, fixation to proper position. Considering that there would be no difference between cohort 1 and 2 in the point of lowermost placement of fat pedicle during the operations, with both techniques enabling fixation in the proper position. Then, it could be assumed that significant differences in aesthetic outcomes between the two cohorts might be associated with higher rate of recurrence in cohort 1, which may result from lesser durability of fixation. A potential risk of recurrence after removal of pulled out thread has been suggested as one of the main drawbacks of the conventional EPS technique, which could be related to insufficient adhesion with adjacent tissue, excessive tension on fat pedicles and accidental external force, such as rubbing of the operative sites during sleeping. Instead, our IF technique allows for rigid and durable fixation of fat pedicle, which could lead to excellent outcomes, suggesting low recurrence, in cohort 2.

Another advantage of this technique over the conventional IF technique is that it provides options to choose between the supraperiosteal and subperiosteal planes. It is known that fat repositioning through the supraperiosteal plane could be relatively faster and easier. Dissection along the supraperiosteal plane could release the orbicularis oculi muscle attachment to the periosteum below arcus marginalis more effectively than subperiosteal dissection, which can allow for improved and more natural effacement of the nasojugal groove. Although it could have higher risks of bleeding and haematoma, it can be avoided using suitable retraction of the orbicularis oculi muscle and sharp electrocautery dissection close to the periosteum. However, despite these potential strengths of supraperiosteal dissection, the conventional IF technique cannot be performed in this plane as there is no anatomic structure to anchor the transferred fat pedicle. It is likely that with the devised needle, easy and secure fixation can be achieved with supraperiosteal dissection besides subperiosteal dissection.

Several limitations of the present study need to be mentioned. Owing to the retrospective study design, effects of other variables that can affect the outcomes, such as comorbidities and medications, could not be assessed. However, our study population consisted of relatively young patients with a mean age of 34 years, who were healthy, which could reduce the risk of confounding effects of those variables. Different time periods between the two cohorts could raise a concern of potential learning curve effect. The relatively inferior outcomes in cases of cohort 1 might be attributable to less skillfulness because they were treated in the former part of the study period. However, the primary surgeons had considerable experience of transconjunctival fat repositioning for tear trough deformity correction before this study and had become sufficiently competent and experienced to perform such procedures, which might have reduced the confounding effect related to learning curve. The patients of private plastic clinics are usually reluctant to visit frequently after operation and tend to be lost to follow-up in early post-operative period. Although we included only patients with follow-up longer than one month and a median follow-up period of the study population was over two months, this would not be sufficient for evaluating long-term efficacy of this procedure. Further well-designed long-term studies would be definitely required to draw more solid conclusions.

## Conclusions

Our results suggest that the new IF technique using EZ-Tcon could have advantages of the conventional IF and EPS techniques, including secure fixation, low relapse rate, elimination of need for thread removal, early recovery, straightforward procedure, short conjunctival incision and optimally lowermost positioning of transposed fat, as summarized in Table 6. This technique could be a safe and reliable method of transconjunctival fat repositioning for the correction of tear trough deformity, with low complication rates and aesthetically satisfactory outcomes.

**Table 6:** Summary of comparison of three fixation techniques

Advantages	Conventional IF	EPS	IF with EZ-Tcon
Stability	V		V
Technical easiness		V	V
Short incision		V	V
Ideal positioning		V	V
Patients convenience	V		V
No conspicuous skin scar	V		V

## Electronic supplementary material

Below is the link to the electronic supplementary material.

Supplementary file1 (MP4 78536 kb)

Supplementary file2 (MP4 39884 kb)

Supplementary file3 (MP4 87323 kb)
